# Special Issue “Biomechanics and Neuromuscular Control of Gait and Posture”

**DOI:** 10.3390/jfmk11040430

**Published:** 2026-10-18

**Authors:** Philippe Gorce

**Affiliations:** 1 International Institute of Biomechanics and Occupational Ergonomics, Avenue du Docteur Marcel Armanet, CS 10121, 83418 Hyères, France; gorce@univ-tln.fr; 2 University of Toulon, CS60584, 83041 Toulon, France

## 1. Introduction

One of the challenges of the 21st century is to enable people to live longer in good health [1]. Maintaining motor functions plays an important role in well-being and quality of life [2]. From this perspective, understanding the mechanisms that lead to a reduction in the effects of ageing, neurodegenerative diseases, and other causes is a major multi-disciplinary scientific challenge. It is therefore important to understand how motor disorders or impairments (or disabilities more generally) modify body biomechanics and motor control of walking or posture. This Special Issue brings together eighteen high-quality publications on recent advances in research into the use of biomechanics and knowledge of neuromuscular control to understand motor alterations, design new therapies, or develop technical aids to restore independence.

## 2. Gait and Postural Control

Walking and maintaining posture are fundamental to preserving functional independence. The development of indices to measure changes in muscular activities or movements of body segments during repetitive tasks is a dynamic scientific area. The work of Padulo et al. suggests using speed-dependent gait variability as a relevant measure to describe a level of bilateral coordination. Kim et al. exploits the use of a kneeling scooter to restore function to the paretic limb. The authors use constraint-induced movement therapy to compensate for limited plantar propulsion in stroke patients.

## 3. Postural Balance

The study of postural balance is widely used in the international literature to understand how motor disorders or alterations modify body biomechanics and the motor control of posture. To compensate for the effects of ageing on the musculoskeletal system, Concha-Cisternas et al. propose to analyze the effects of neuromuscular training on postural balance, physical performance, and muscle strength in elderly women. Alves et al. studied the body dynamics of subjects with achondroplasia. The authors analyzed the variability of postural oscillations in the standing posture of subjects with natural versus altered growth. Floessel et al. proposed assessing postural control using the center of pressure in the seated position. The author’s aim was to propose an alternative diagnostic method for quantifying neuromuscular control. Postural control is also used in sport to describe the strategies employed by the spino-pelvic complex in handstand. For example, Eyssartier et al. studied sagittal alignment and balance of the spine using radiographic images.

## 4. New Therapies

The development of new therapies is also contributing to scientific knowledge of the underlying mechanisms linked to motor impairment. For example, the work of Bikhetava et al. examines the effect of transcutaneous stimulation of the cervical spinal cord on postural stability in healthy participants. The authors used the center of pressure and frequency spectrum of the statokinesigram with open or closed eyes on various surfaces. Brodie et al. studied age-related differences between healthy young adults and older adults with no history of falls in the stumble correction responses evoked by electrical stimulation of the superficial peroneal cutaneous nerve during walking. To improve motor function, Christofoletti et al. use whole-body vibration to test the acute effects of a single vibration session on mobility and postural control in elderly people. Branney et al. studied the underlying pathophysiology of neck pain. The authors measured the prevalence of intervertebral disc degeneration and continuous cervical intervertebral motion in patients with sub-acute or chronic neck pain and in gender-matched healthy controls. Centemeri et al. studied the early detection of femoro-acetabular impingement and the progression of pathology over time. The neural-musculoskeletal system of affected subjects was assessed using two modalities: the balance test and the MuscleLab test, compared with the flexion–abduction–external rotation and flexion–adduction–internal rotation tests.

## 5. Muscle Injuries

Muscle damage can lead to motor impairment. Hamstring injuries are common in sport. To reduce their incidence, Sos-Tirado et al. propose to compare the short-term effects of proprioceptive neuromuscular facilitation stretching (with and without electrical stimulation) on hamstring extensibility and vertical jumping ability in young basketball players. Palermi et al. propose using artificial intelligence to predict and prevent muscular injuries, particularly in football.

## 6. Muscle Strength

Knowledge of muscle force amplitudes is an important element in understanding motor disorders or alterations. Dufner et al. examined the differences in force production and pain in participants between a maximal voluntary isometric squat contraction performed with a belt or a full harness. Ortega et al. investigated the effects of sustained anchored forearm isometric flexion tasks on patterns of fatigue-induced changes in torque and neuromuscular responses, time to task failure, and performance fatigability. Heller et al. compared the strength–velocity relationship during cycling and arm cranking in men and women.

## 7. Musculoskeletal Disorders and Diseases

Musculoskeletal disorders and disease can be the result of inappropriate occupational or leisure activity, with major consequences for functional autonomy. Thus, Gorce et al. proposed a literature review and meta-analysis to study the overall prevalence of work-related musculoskeletal disorders in nurses in Europe and by body area. Bruscemi et al. evaluated the existing literature on sports vision, paying particular attention to the effects of visual training and its application in different sports and in rehabilitation and prevention contexts.

## 8. Conclusions

In summary, the publications in this Special Issue constitute a significant advance in the field of biomechanics and knowledge of neuromuscular control for understanding motor alterations due to musculoskeletal disorders and diseases. It also clearly shows that this multi-disciplinary scientific theme is very dynamic and that it affects a wide range of fields (such as sport, health, rehabilitation, ergonomics, etc.). Its many connections with other fundamental sciences such as engineering, medicine, neuroscience, and the humanities mean that it can also benefit from technical innovations and technological advances in communication, robotics, and sensors. This theme is therefore set to evolve quickly, considering the increasingly connected world we live in, while preserving people’s health, well-being, and quality of life.

We would like to express our sincere gratitude to all the authors and reviewers who contributed to this Special Issue and to the team at the *Journal of Functional Morphology and Kinesiology* for their invaluable help and support.

## References

[1] Organisation Mondiale de la Santé (2015). Rapport Mondial sur le Vieillissement et la Santé.

[2] Ge Y., Zhao W., Zhang L., Zhao X., Shu X., Li J., Liu Y. (2024). Correlation between motor function and health-related quality of life in early to mid-stage patients with Parkinson disease: A cross-sectional observational study. Front. Aging Neurosci..

